# Antineutrophil Cytoplasmic Antibody (ANCA)-Positive Eosinophilic Granulomatosis With Polyangiitis Presenting With Refractory Asthma, Mononeuritis Multiplex, and Cutaneous Vasculitis: A Case Report

**DOI:** 10.7759/cureus.110239

**Published:** 2026-06-04

**Authors:** Khaled M Malkawi, James Beynon, Hitasha Rupani

**Affiliations:** 1 NIHR Southampton Clinical Research Facility, University Hospital Southampton NHS Foundation Trust, Southampton, GBR; 2 Rheumatology, University Hospital Southampton NHS Foundation Trust, Southampton, GBR; 3 Respiratory Medicine, University Hospital Southampton NHS Foundation Trust, Southampton, GBR

**Keywords:** adult-onset asthma, anca associated vasculitis, atypical egpa, eosinophilia, eosinophilic granulomatosis with polyangiitis, mononeuritis multiplex

## Abstract

Eosinophilic granulomatosis with polyangiitis (EGPA) is a rare antineutrophil cytoplasmic antibody (ANCA)-associated small-vessel vasculitis characterised by asthma, eosinophilia, and eosinophil-rich tissue inflammation. Early diagnosis is often difficult because initial features may resemble uncontrolled asthma or other eosinophilic lung diseases. We report a 66-year-old man with adult-onset asthma who presented with four months of progressive dyspnoea, constitutional symptoms, peripheral neuropathy, and cutaneous vasculitis. Investigations showed marked eosinophilia (19.1 × 10⁹/L), raised inflammatory markers, elevated creatine kinase and lactate dehydrogenase, and markedly elevated IgG4. Cross-sectional imaging showed calcified mediastinal and hilar lymph nodes without pulmonary infiltrates. Extensive infectious and haematological investigations were unrevealing. Anti-myeloperoxidase antibodies were positive, and skin biopsy confirmed small-vessel vasculitis with eosinophilic infiltration. A diagnosis of ANCA-positive EGPA was made. The patient was treated with high-dose corticosteroids, but symptom recurrence during steroid taper prompted escalation to intravenous cyclophosphamide for organ-threatening neurological involvement. This case highlights the importance of considering EGPA in patients with refractory asthma, eosinophilia, and systemic features even in the absence of pulmonary infiltrates. Early recognition and multidisciplinary management are essential to reduce irreversible organ damage.

## Introduction

Pulmonary eosinophilic disorders are a heterogeneous group of diseases with the unifying characteristic of eosinophilia within the airways and/or lung parenchyma. They include common conditions such as asthma and rarer diseases such as eosinophilic granulomatosis with polyangiitis (EGPA), and can range in severity from asymptomatic to life-threatening [1-4].

One of the differential diagnoses for pulmonary eosinophilia is eosinophilic granulomatosis with polyangiitis (EGPA; formerly known as Churg-Strauss syndrome), which is a rare small-vessel vasculitis that occurs in patients with asthma and eosinophilia and is histologically characterised by tissue eosinophilia, necrotizing vasculitis and eosinophil-rich granulomatous inflammation [5-7].

EGPA is a rare disease with an annual global incidence rate of 2.15 cases per million people per year and a prevalence of 34.44 cases per million people-years, with an average age at diagnosis of 57 years [8,9].

The diagnosis of EGPA remains challenging and is often delayed or missed due to its rarity, heterogeneous clinical phenotype, and overlap with other eosinophilic and small-to-medium vessel vasculitic disorders. Typical manifestations include asthma, sinonasal disease, peripheral eosinophilia, neuropathy, and variable extrapulmonary organ involvement, necessitating careful clinical assessment and exclusion of vasculitis mimics [9].

We report a case of antineutrophil cytoplasmic antibody (ANCA)-positive EGPA presenting with refractory adult-onset asthma, marked eosinophilia, neuropathy, and biopsy-proven cutaneous vasculitis, highlighting the diagnostic difficulty of EGPA in the absence of pulmonary infiltrates.

## Case presentation

A 66-year-old man with adult-onset asthma was admitted with a four-month history of progressive dyspnoea. He had been reviewed in primary care, and his asthma inhalers were up-titrated on several occasions without clinical benefit. Two months prior to admission, he started to develop constitutional symptoms including profound fatigue, night sweats, malaise, and 4 kg weight loss. In addition, he reported pins and needles in his hands as well as worsening sinonasal congestion. He reported no travel outside the United Kingdom in the preceding five years. In the months prior to hospital admission, he had also received two courses of oral steroids from his primary care physician to treat asthma exacerbations.

Physical examination showed tender cervical and inguinal lymphadenopathy as well as bilateral leg swelling associated with an itchy rash consisting of non-blanching palpable purpuric papules with surrounding violaceous macules (Figure 1). Neurological examination demonstrated left-sided proximal weakness, severe neuropathic pain in the hands and feet, left foot drop with weak ankle dorsiflexion, and absent ankle jerk reflex.

**Figure 1:**
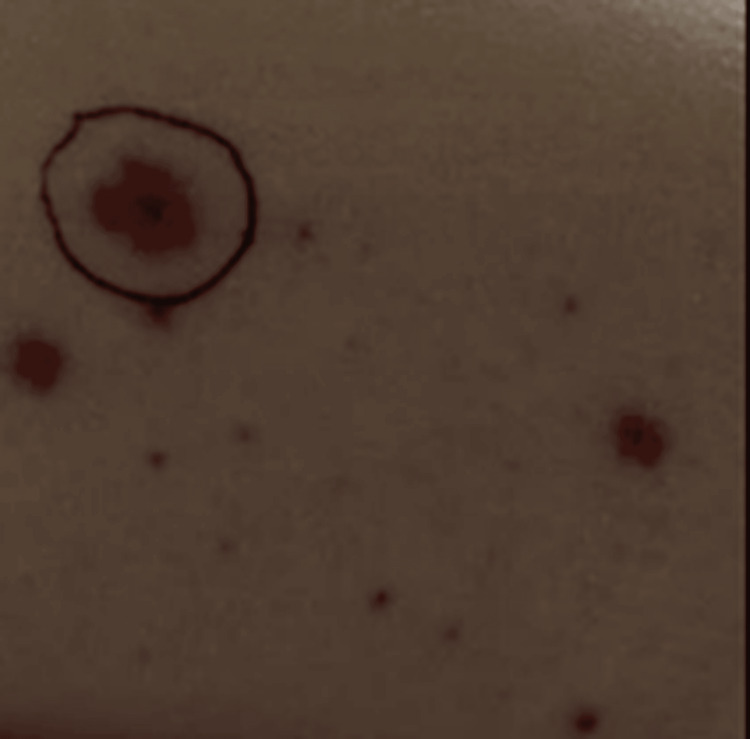
Palpable purpuric rash over the lower limbs consistent with cutaneous vasculitis. The ink-drawn ring is a clinical site marker, and not part of the rash.

On admission, laboratory investigations demonstrated a markedly elevated white cell count (28.4 × 10⁹/L), characterised by significant eosinophilia with an absolute eosinophil count of 19.1 × 10⁹/L. Inflammatory markers were also raised, including C-reactive protein (CRP) level of 37 mg/L and an erythrocyte sedimentation rate (ESR) of 33 mm/h. Lactate dehydrogenase (LDH) was elevated at 837 U/L. Relevant admission laboratory values are summarized in Table 1.

**Table 1:** Admission laboratory investigations Hb: Haemoglobin; WBC: White blood cell; LDH: Lactate dehydrogenase; TSH: Thyroid-stimulating hormone; CRP: C-reactive protein; ESR: erythrocyte sedimentation rate; eGFR: estimated glomerular filtration rate; NT-Pro BNP: N-terminal pro-B-type natriuretic peptide.

Blood test	Value	Unit	Normal range
Hb	118	g/L	130 - 170
WBC	28.4	×10⁹/L	4.0 - 10.0
Platelets	315	×10⁹/L	150 - 410
Neutrophils	6.6	×10⁹/L	2.0 - 7.0
Eosinophils	19.1	×10⁹/L	0.0 - 0.5
Calcium (adjusted)	2.15	mmol/L	2.20 - 2.60
Alkaline Phosphatase	130	U/L	30 - 130
Albumin	29	g/L	35 – 50
Troponin I	7	ng/L	0 - 18
Creatine Kinase (CK2)	816	U/L	40 - 320
Ferritin	428	µg/L	23.9 - 336
LDH	837	U/L	208 - 378
TSH	1.90	mU/L	0.38 - 5.3
CRP	37.00	mg/L	0 - 5
ESR	33	mm/h	1 – 14
Sodium	134	mmol/L	133 - 146
Potassium	4.30	mmol/L	3.5 - 5.3
Urea	3.8	mmol/L	2.5 - 7.8
Creatinine	65	µmol/L	59 - 104
eGFR	>90	mL/min/1.73 m²	
Immunoglobulin G4	9.240	g/L	<1.3
NT-Pro BNP	217	ng/L	20 - 200
Complement C3	1.49	g/L	0.75 – 1.65
Complement C4	0.43	g/L	0.14 – 0.54

A blood film showed eosinophilia with no blast cells. Cross-sectional imaging of his neck, chest, abdomen and pelvis was performed and showed multiple calcified mediastinal and left hilar lymph nodes with no lung parenchymal changes and no cervical lymphadenopathy (Figure 2).

**Figure 2:**
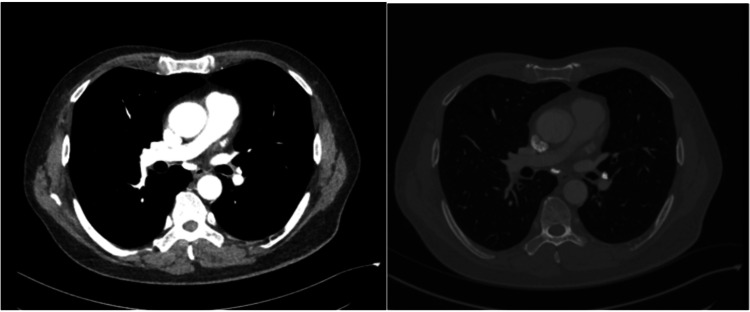
CT imaging demonstrating calcified mediastinal and left hilar lymph nodes without focal pulmonary infiltrates or other parenchymal abnormalities.

Given the multisystem presentation, a broad differential diagnosis was considered, including infection, haematological malignancy, and connective tissue disease. Infectious investigations, including serology for schistosomiasis, toxocara, toxoplasma, and strongyloides, as well as tests for syphilis, cytomegalovirus (CMV), Epstein-Barr virus (EBV), antistreptolysin O, faecal pathogen PCR, faecal microscopy, and Aspergillus serology, were negative. QuantiFERON testing was indeterminate and was reviewed by the infectious diseases team who concluded that it was due to previous TB contact in childhood. Autoimmune testing showed positive ANCA with elevated anti-myeloperoxidase antibodies (53 U), while antinuclear antibody (ANA) and the extended connective tissue disease screen were negative.

He was reviewed by the respiratory team 48 hours after admission, and the combination of adult-onset asthma, marked eosinophilia, constitutional symptoms, neuropathy, and cutaneous vasculitis raised a strong suspicion for EGPA. As the team was formulating his treatment regimen, his vasculitis screen was reported as showing ANCA positivity with elevated anti-myeloperoxidase (MPO) antibodies (53 U). A diagnosis of ANCA-positive EGPA was confirmed. He remained under the joint care of respiratory and rheumatology teams after his diagnosis.

Further investigations supported multisystem EGPA involvement. Nerve conduction studies showed patchy attenuation of sensory and motor amplitudes in the lower limbs, and electromyography demonstrated changes consistent with vasculitic neuropathy with superimposed mononeuritis multiplex. Echocardiography and cardiac MRI showed no evidence of eosinophilic cardiomyopathy. CT of the sinuses demonstrated severe left-sided paranasal sinus mucosal thickening. Urinalysis and urinary albumin-creatinine ratio were normal. Skin punch biopsy from the lower limb rash confirmed small-vessel vasculitis with leukocytoclasis, red cell extravasation, eosinophilia, and vessel wall IgA deposition.

Once a clinical diagnosis of eosinophilic granulomatosis with polyangiitis (EGPA) was established, the patient was commenced on intravenous methylprednisolone 1 g once daily for two days, followed by oral prednisolone 40 mg daily with a tapering plan of 10 mg every two weeks. He had a rapid improvement in symptoms, including resolution of lower limb weakness. However, when prednisolone was reduced to 30 mg daily two weeks later, he developed increased fatigue and lethargy. The prednisolone dose was therefore escalated to 50 mg daily, with an aim to taper according to clinical response, although this adjustment did not result in significant symptomatic improvement.

Given the suboptimal response, the need for a second induction agent was extensively discussed among the Respiratory, Rheumatology, and wider multidisciplinary teams. Therapeutic options considered included rituximab, benralizumab, mepolizumab, and cyclophosphamide. Ultimately, the teams agreed to initiate cyclophosphamide at 15 mg/kg fortnightly as first-line induction therapy, with plans to reassess after three doses and to consider the addition of benralizumab if prednisolone tapering remained unsuccessful.

After six cycles of cyclophosphamide, the patient had marked improvement in dyspnoea and neuropathic symptoms. Benralizumab was subsequently commenced, and prednisolone was tapered to 15 mg daily. His asthma was better controlled, with an ACQ score of 1.66 and FeNO of 59 ppb, although mild residual neurological symptoms persisted.

## Discussion

Eosinophilic granulomatosis with polyangiitis (EGPA) is a rare multisystem disorder that can present insidiously, often after a long history of asthma and rhinosinusitis. The diagnosis in this case was challenging due to the initial presentation mimicking poorly controlled asthma, a common scenario in EGPA where eosinophilic inflammation precedes overt vasculitis by months or even years [6]. The presence of progressive constitutional symptoms, marked eosinophilia, neuropathy, and cutaneous vasculitis prompted consideration of systemic disease.

EGPA is considered as a rare disease; a systematic review showed that the incidence of EGPA is 2.15 cases per million persons per year, and prevalence of 34.44 cases per million people, with country-specific studies having less than 8 cases per million persons per year and prevalence less than 60 cases per million [8]. A UK-based database study reported that the incidence and prevalence of EGPA almost doubled between 2005 and 2019 [9].

EGPA is an ANCA-associated vasculitis that is commonly linked to anti-myeloperoxidase antibodies. However, up to 70% of patients may be ANCA-negative [10]. Patients who are ANCA-negative are more likely to have features of cardiac and gastrointestinal involvement as well as pulmonary infiltrates, whereas ANCA-positive patients have a higher frequency of clinical vasculitis manifestations such as peripheral neuropathy or renal involvement, but less frequently have cardiomyopathy [11,12]. The patient fulfilled the characteristic clinical and laboratory features of ANCA-positive EGPA, including adult-onset asthma, peripheral eosinophilia, and histopathological evidence of small-vessel vasculitis with eosinophilic infiltration without cardiac or renal involvement.

Vessel-wall IgA deposition on skin biopsy is classically regarded as the histopathological hallmark of IgA vasculitis, rather than a typical feature of EGPA [5]. In contrast, EGPA vasculitis is usually characterised histologically by eosinophil-rich necrotising small to medium vessel vasculitis and granulomatous inflammation [5]. Therefore, the presence of IgA deposition in our case is notable, as it introduces an unusual histopathological finding that may mimic or overlap with IgA vasculitis, while the overall clinical picture of adult-onset asthma, marked eosinophilia, ANCA positivity, neuropathy, and eosinophilic cutaneous vasculitis remains most consistent with EGPA.

The differential diagnosis of pulmonary eosinophilia is broad. Infectious causes such as parasitic infestations were excluded through serology and stool examination. Aspergillus serology was negative, excluding allergic bronchopulmonary aspergillosis (ABPA) as a differential. Haematological malignancies were also considered given the weight loss and constitutional symptoms, but were excluded given the absence of any significant lymphadenopathy or organomegaly on cross-sectional imaging. Given his positive ANCA status, the haematology team did not feel a bone marrow biopsy or any additional cytogenetic testing was needed. This approach aligns with current European Respiratory Society (ERS) recommendations for stepwise evaluation of pulmonary eosinophilia, emphasizing exclusion of secondary causes before establishing a diagnosis of EGPA [6].

Treatment of EGPA depends on organ involvement. As our patient had organ-threatening involvement, he was treated with high-dose intravenous steroids and intravenous cyclophosphamide. This strategy is consistent with contemporary guidance for ANCA-associated vasculitis, which supports cyclophosphamide or rituximab for severe organ-threatening disease [13].

Cyclophosphamide is a cytotoxic alkylating agent of the nitrogen mustard class that cross-links DNA, thereby inhibiting DNA and RNA synthesis and function. It remains within the treatment algorithm of many EGPA guidelines. This was supported by a prospective clinical trial in 48 EGPA patients with a Five-Factor Score (FFS) ≥ 1, which concluded that 12 cyclophosphamide pulses were better able to control severe EGPA than a 6-pulse regimen [14].

IL-5 has a crucial role in promoting eosinophil proliferation, differentiation, migration to tissues, and prolonged survival by inhibiting apoptosis [15]. Multiple studies have shown the effectiveness of mepolizumab in the treatment of EGPA in reducing reliance on corticosteroids and immunosuppressants [16-18]. In the MIRRA study, 28% of participants in the mepolizumab treatment group had ≥24 weeks of remission versus 3% in the placebo group [17]. The MANDARA study evaluated the efficacy and safety of benralizumab and mepolizumab and demonstrated that benralizumab is noninferior to mepolizumab for the induction of remission in patients with relapsing or refractory EGPA (59% of participants in the benralizumab group had remission at weeks 36 and 48 compared to 56% in the mepolizumab group, p=0.73 for superiority, 95% CI) [19]. A recent three-year real-world study demonstrated long-term effectiveness and safety of benralizumab in the treatment of EGPA, with clinical remission rates rising from 39.4% (95% CI: 22.9-57.9) at three months to 65.0% (95% CI: 40.8-84.6) at 36 months (p<0.001), and corticosteroid dependence reducing from 90.9% to 15.4% in 33 patients with EGPA [20].

Neurological complications, as seen in this case, are well-recognised but less common manifestations of EGPA. Central nervous system involvement, often due to small-vessel vasculitis or thromboembolic phenomena, carries a poor prognosis and can result in permanent disability [15].

This case illustrates the multisystemic nature of EGPA and highlights the diagnostic importance of considering vasculitis in patients with deteriorating asthma, systemic symptoms, and eosinophilia. Early recognition and multidisciplinary management are key to improving long-term outcomes.

A limitation of this report is that it describes a single patient experience, so treatment response and disease course may not be generalisable.

## Conclusions

EGPA should be considered in patients with refractory adult-onset asthma, marked eosinophilia, and systemic manifestations such as neuropathy or vasculitic rash, even when chest imaging does not show pulmonary infiltrates. This case highlights the value of early multidisciplinary assessment, tissue confirmation when feasible, and timely escalation to remission-induction therapy in organ-threatening disease.
